# Pain Catastrophizing Thoughts Explain the Link Between Perceived Caregiver Responses and Pain Behaviors of Patients With Chronic Musculoskeletal Pain

**DOI:** 10.3389/fpsyg.2020.01386

**Published:** 2020-07-03

**Authors:** Somayyeh Mohammadi, Fatemeh Alinajimi, Nasrin Esmaeilian, Mohsen Dehghani, Ali Khatibi

**Affiliations:** ^1^Department of Occupational Science and Occupational Therapy, Faculty of Medicine, The University of British Columbia, Vancouver, BC, Canada; ^2^Family Research Institute, Shahid Beheshti University, Tehran, Iran; ^3^Department of Clinical Psychology and Health, Shahid Beheshti University, Tehran, Iran; ^4^Neuroepidemiology Unit, The Melbourne School of Population and Global Health, The University of Melbourne, Parkville, VIC, Australia; ^5^Centre of Precision Rehabilitation for Spinal Pain, School of Sport, Exercise and Rehabilitation Sciences, College of Life and Environmental Sciences, University of Birmingham, Birmingham, United Kingdom; ^6^Centre for Human Brain Health, University of Birmingham, Birmingham, United Kingdom

**Keywords:** pain, caregiver, pain behavior, catastrophizing, responses

## Abstract

**Purpose:**

Caregivers’ responses to pain behaviors of patients with chronic pain have an essential role in how patients perceive their pain condition. The current study investigated the mediating role of pain catastrophizing on the link between perceived caregiver responses and patient pain behaviors.

**Materials and Methods:**

The sample of this cross-sectional study consisted of 200 patients with chronic pain (mean of age = 44.6; 71.5% were female). Participants responded to measures assessing their perception of their caregiver responses to their pain, their pain catastrophizing thoughts, and their pain behaviors.

**Results:**

The mediation analyses showed that perceived distracting responses were negatively related to pain catastrophizing level in patients, which in turn was positively associated with expressing pain behaviors. Besides, perceived caregiver negative responses were positively associated with catastrophizing thoughts, which in turn was positively related to expressing pain behaviors.

**Conclusion:**

Patients’ perceptions regarding how their caregiver responds to their pain condition can be related to their thoughts about their pain and how they react to their pain situation. Investigating the external sources that might have an impact on patients’ reactions to their pain, especially when those external sources are caregivers who, in most situations, are with the patients for a prolonged duration, is essential.

## Introduction

Chronic pain resulting from musculoskeletal conditions never occurs in isolation. Studies on chronic pain have been shown that family factors, including the relationship with other family members and their reactions to pain, have substantial impacts on pain intensity and pain-related disability (Smith et al., 2019). Chronic musculoskeletal pain is an invisible disability, and in most cases, observers, especially family caregivers, do not have any visible physical clues (for example, an injury wound) to help them understand the pain experience and provide the care that patients with chronic pain need. Without having any physical cues to rely on, it is more likely that patients with chronic pain (unlike other patients such as patients with cancer) receive less support from their family caregivers. Therefore, considering that about 20–25% of adult population experienced musculoskeletal pain at some point in their life (Goldberg and McGee, 2011) and chronic musculoskeletal conditions including osteoarthritis and spinal disorders are among the leading causes of mobility impairment in adults (Whittington et al., 2019), it is essential to investigate the interactions among patients with chronic pain and their family caregivers and to explore how these interactions are related to patients’ pain experience.

To understand the patients’ pain-related experience, family caregivers may rely on pain behaviors. Pain behaviors, such as distorted walking, aim to reduce pain intensity or prevent further injury (Kerns et al., 1990; Martel et al., 2010). However, besides their protective nature, pain behaviors can convey pain intensity to the observers, including family caregivers (Kerns et al., 1990). The ability to discover patients’ pain behaviors is crucial for caregivers to provide appropriate and timely support (Boerner et al., 2013; Mohammadi et al., 2015). Caregivers’ responses to pain behaviors including solicitous (e.g., taking over daily activities), distracting (e.g., encourage the patient to watch TV), and negative responses (e.g., leaving the room) are found to be related to the pain behaviors expressed by the patients (Williamson et al., 1997; Mohammadi et al., 2017b). According to the cognitive–behavioral conceptualization of pain, perceptions of caregivers’ responses are related to the number of pain behaviors expressed by patients (Cano et al., 2000). For example, it has been shown that caregivers’ solicitous and negative responses are related to expressing more pain behaviors (Flor et al., 1987; Romano et al., 2000; Raichle et al., 2011; Edmond and Keefe, 2016), while distracting responses are expected to be related to lower levels of pain behaviors (Romano et al., 2000). While the link between caregiver responses to pain and pain behaviors has gained some supports, the pathway through which the caregivers’ responses are associated with pain behaviors expressed by patients is not clear.

One of the factors that can impact patients’ pain behaviors is pain catastrophizing that is an exaggerated negative orientation toward a painful experience (Sullivan et al., 1995). Rumination about pain is a component of pain catastrophizing, e.g., “I keep thinking this is terrible,” as well as feeling helpless, e.g., “I thought it was never going to get better,” and magnifying the pain experience and its consequences, e.g., “I think of other painful experiences” (Sullivan et al., 1995). Research has shown a positive association between pain catastrophizing and pain behaviors (Flor et al., 1987; Thibault et al., 2008; Gauthier et al., 2011; Mohammadi et al., 2017a). Some studies suggest that psychological factors such as depression and anxiety are among the strongest predictors of pain catastrophizing (Leeuw et al., 2007; Park et al., 2016). Considering that pain catastrophizing cognitions are often accompanied by pain behaviors and the communicative function of pain behaviors (Badr and Shen, 2014), it can be suggested that social context may also influence pain catastrophizing in patients with chronic pain. Specifically, caregivers’ *solicitous* responses that demand patients to take rest or stop their current activities may convey the notion to patients that there is a serious threat and therefore increase the pain catastrophizing thoughts (Mohammadi et al., 2017a). Caregivers’ *distracting* responses, on the other hand, can divert patients’ attention from pain to other stimuli and hence result in lower pain catastrophizing thoughts, which is in line with previous studies that emphasized on the role of distraction techniques and disengagement from the pain stimuli in reducing pain catastrophizing level in patients (Van Damme et al., 2004; Van Ryckeghem et al., 2012). Finally, caregivers’ *negative responses* may imply that help and support may not be available when patients are in need, which can contribute to patients’ helplessness, followed by increased catastrophizing levels (Mohammadi et al., 2017a). While previous research provides some evidence that there is a link between caregivers’ responses to pain and pain catastrophizing, it is not yet clear whether pain catastrophizing in patients can mediate the link between their perception of their caregivers’ responses to pain and their pain behaviors. Hence, the goal of the current study was to understand how patients’ perception of caregiver responses was associated with the pain behaviors expressed by patients.

**FIGURE 1:**
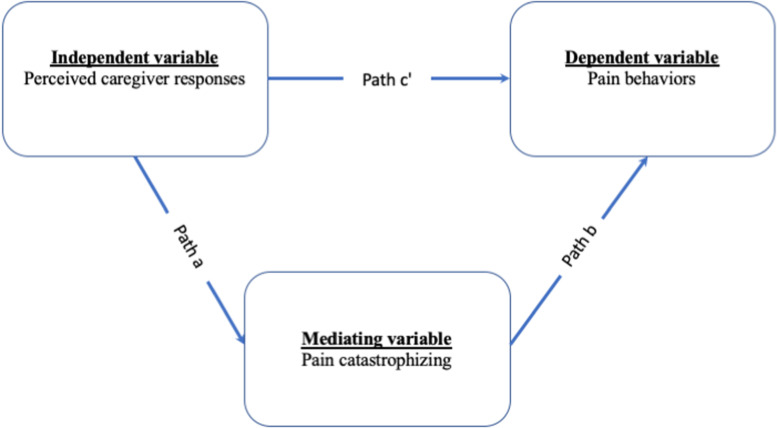
The hypothetical mediation model.

Therefore, the present study hypothesized that (1) perceiving more caregivers’ solicitous responses is related to higher levels of pain catastrophizing in patients. In turn, higher pain catastrophizing is related to more pain behaviors; (2) perceiving more caregivers’ distracting responses is related to lower levels of pain catastrophizing in patients. In turn, lower levels of pain catastrophizing are related to fewer pain behaviors; and (3) perceived more caregivers’ negative responses are related to higher levels of pain catastrophizing in patients. In turn, higher pain catastrophizing is related to more pain behaviors. Figure 1 shows the hypothetical mediation model.

## Materials and Methods

### Procedure and Participants

The data were collected from four physiotherapy clinics in Esfahan, Iran. Nurses and front desk staff at these clinics identified the eligible patients and notified one of the researchers who were present at the clinic (FA) and also invited the patients to participate in the study. First, the researcher explained the study and checked the eligibility of the patients. Inclusion criteria were having musculoskeletal pain on most days for at least 3 months, being over 18 years of age, fluent in reading and writing in Persian, having at least one caregiver, and living with their caregiver. Exclusion criteria were declaring a medical history of major psychiatric disorder, concussion, or head injury, and declaring current drug and alcohol abuse. Furthermore, patients with other illnesses and disorders who experienced pain as one of their symptoms such as patients with cancer, AIDS, shingles, stomach ulcers, and nerve damage were excluded due to the difference in the causes and symptom presentations of these disorders with the causes and symptom presentations in chronic musculoskeletal pain disorders. Two-hundred and fifty patients were eligible to participate in the study. Twenty patients did not agree to participate in the study. The main reasons were that they declared intense pain, and they did not seem in good health to fill out the questionnaires. Twenty-three patients returned the questionnaires without answering the questions. Seven patients fill out <40% of the questions. Therefore, their data were removed from the final dataset. Finally, the data of 200 patients were analyzed.

### Ethics Statement

All participants declared consent before participating in this study. The study was approved by the Psychology Department’s Research Ethics Board.

### Measures

#### Demographics Characteristics

Patients reported sex, age, duration of pain, usage of analgesic medicines, and pain location (e.g., back, leg, knee, neck).

#### Perceived Caregiver Responses

To assess perceived caregiver responses, the significant other section of the West Haven-Yale Multidimensional Pain Inventory (WHYMPI) was used (Kerns and Turk, 1985; Kerns et al., 1985). This section had 14 self-report items that assessed three subscales: solicitous responses (e.g., “asks me who he/she can help”), distracting responses (e.g., “involves me in activities”), and punishing responses (e.g., “expresses frustration at me”). Participants rated the frequency of each item on the seven-point Likert scale (0 = never to 6 = very often). A higher average score (ranging from 0 to 6) indicated more perceived caregiver responses on each subscale. The significant other section of the WHYMPI has acceptable reliability and validity (Kerns et al., 1985). The internal reliability of the solicitous, distracting, and punishing responses in the current sample was 0.76, 0.82, and 0.72, respectively.

#### Patient’s Pain Behaviors

Pain behaviors were measured by the Pain Behavior Checklist (PBCL; 23). The PBCL had 17 items (e.g., “move extremely slowly,” “become irritable,” “talk about my pain problem,” “clench my teeth”). Patients reported the estimation of their pain behaviors on a seven-point Likert-type scale (0 = never, 6 = very often). A higher average score showed higher levels of pain behaviors. It was shown that the PBCL has acceptable reliability and validity (Kerns et al., 1991). In the current sample, the Cronbach’s alpha of the total score of the PBCL was 0.88.

#### Pain Catastrophizing

To assess pain catastrophizing of patients, the Pain Catastrophizing Scale (PCS) was used (Sullivan et al., 1995). The PCS is a 13-item self-report scale evaluating catastrophic thoughts or feelings in relation to pain experiences (e.g., “I can’t seem to keep it out of my mind”) on a five-point Likert-scale ranging from 0 (not at all) to 4 (always). A higher average score indicated more pain catastrophizing. In the current study, the internal consistency for pain catastrophizing was 0.90. The PCS had good psychometric properties (Osman et al., 2000; Dehghani et al., 2014; Akbari et al., 2016).

### Statistical Plan

The association between perceived caregiver responses to pain, pain catastrophizing, and pain behaviors were measured by Pearson correlations (please see Table 1). Mediation analyses were evaluated with the PROCESS (Hayes, 2013). To test the mediating role of a denoted mediator (i.e., patient pain catastrophizing) in the relation between an independent variable (i.e., perceived caregiver responses) and dependent variable (i.e., pain behaviors), the model 4 of the PROCESS was used. The total effect of an independent variable on a dependent variable was shown by weight *c* and composed of the direct effect of the independent variable on the dependent variable (weight *c’*) and the indirect effect of the independent variable on a dependent variable through a denoted variable (weight *ab*). In addition, the effect of an independent variable on a defined mediator was presented by weight *a*. Lastly, weight *b* expressed the effect of a denoted mediator on a dependent variable while excluding the effect of the independent variable. In the mediation analyses, we used a bootstrap test (with 5,000 resample) to assess the significance of the indirect effect (Preacher and Hayes, 2004).

**TABLE 1:** Means, standard deviations (*SDs*), and correlations among all the main variables in the study.

Main variables in the study	Mean	*SD*	1	2	3	4	5
1. Perceived caregiver solicitous responses	25.89	7.69	1				
2. Perceived caregiver distracting responses	13.12	6.72	0.56**	1			
3. Perceived caregiver negative responses	5.34	5.71	−0.26**	−0.32**	1		
4. Pain catastrophizing	28.07	10.73	0.05	−0.18*	0.33**	1	
5. Pain behaviors	3.09	1.26	0.16*	0.04	0.28**	0.53**	1

*p < 0.05; **p < 0.01.

It should be noted that based on the traditional view of mediation analyses, total effect (*c*) should be significant to be able to calculate the indirect effect (path *ab*; Baron and Kenny, 1986). However, currently, there is a substantial agreement among statisticians that the significance level of total effect (*c*) is not a prerequisite for mediation analysis. Therefore, even when the total effect is not significant, but the indirect effect (path *ab*) is significant, it is still valid to call the observed indirect effect, a mediating effect (Hayes, 2009). Based on Hayes (2009), two main reasons for this conclusion are as follows: (a) the total effect is the sum of direct (*c*′) and indirect effect (*ab*). Therefore, in some cases, when the direct effect is positive and indirect effect is negative (or the other way around), they would neutralize each other, and the total effect will potentially become non-significant; (b) the traditional step approach to calculate mediation analyses indicated that step 1 for conducting a mediation analysis is calculating the association between *X* and *Y*. Based on the traditional model, if no association is found in step 1, the mediation analysis should be stopped. However, currently, statisticians consider this step as illogical because there are many new approaches to calculate the indirect effect directly (without calculating the total effect).

## Results

### Descriptive Characteristics

Our samples consisted of 200 patients with chronic musculoskeletal pain. About two-thirds of the patients were female (71.5%; *n* = 143) and the rest (28.5%; *n* = 57) were male. The mean age of the patients was 44.6 years (*SD* = 13.8). The average duration for pain was 61.21 months (*SD* = 72.9). About half of the patients were taking analgesic medicines (61.5%). The current study asked patients to specify their relationship with their caregivers. Almost half of the caregivers were spouses (46.5%; *n* = 93). Thirty-five percent of patients identified their daughters as their caregiver (*n* = 70). Ten percent of patients (*n* = 20) identified their mothers as their caregivers (offspring as the patient, mother as the caregiver), 3.5% of the patients identified their sons as their caregivers (*n* = 7), 3% (*n* = 6) indicated that their sister is their caregiver, and 2% (*n* = 4) identified other family members (e.g., daughter-in-law) as their caregiver. In addition, based on patients’ report, 71.5% (*n* = 143) of caregivers were women and 28.5% (*n* = 57) were men. In addition, the mean age of caregivers was 37.0 (*SD* = 13.3). Six percent of the patients (*n* = 12) had back pain, 8.0% (*n* = 16) had pain in legs, 16.0% (*n* = 32) experienced pain in knees, 2.0% (*n* = 4) had pain in neck, 7.5% (*n* = 15) experienced pain in two locations, and 60.5% (*n* = 121) had pain in more than two locations.

Specifically, the results of the Pearson correlation analyses showed that caregiver perceived solicitous responses and pain behaviors of patients were significantly and positively correlated (*p* < 0.05). Besides, caregiver perceived distracting responses and patient pain catastrophizing were significantly but negatively correlated (*p* < 0.05). Finally, there were significant and positive correlations among perceived caregiver negative responses, patient pain behaviors, and patient pain catastrophizing (*p* < 0.01). Table 1 presents the means and standard deviations among all the variables in the study.

### Mediating Analyses

#### Perceived Solicitous Responses as the Predictor and Pain Behaviors as an Outcome

Results showed that the total effect of perceived caregiver solicitous responses on patient pain behaviors (weight *c*) was significant, *F*(1,198) = 0.594, *p* < 0.001, *R*^2^ = 0.002. The direct effect of perceived caregiver solicitous responses on patient pain behaviors (weight *c’*) was also significant [*b* = 0.074, *t*(198) = 0.771, *p* < 0.001]. However, the indirect effect (weight *a^∗^b*) did not reach a significant level (coefficient = 0.004, 95% bootstrap CI = −0.007–0.016), showing the lack of significant mediation role of patient pain catastrophizing in the link between perceived caregiver solicitous responses on patient pain behaviors.

#### Perceived Distracting Responses as the Predictor and Pain Behaviors as an Outcome

The total effect of perceived caregiver distracting responses on patient pain behaviors (weight *c*) was not significant, *F*(1,198) = 6.01, *p* > 0.001, *R*^2^ = 0.032. The direct effect (weight *c*′) did reach to a significant level [*b* = −0.289, *t*(198) = −2.453, *p* = 0.015]. Importantly, indirect effect (weight *a^∗^b*) was significant (coefficient = −0.019, 95% bootstrap CI = −0.035 to −0.003), indicating a significant mediating effect of patient pain catastrophizing on the relationship between perceived distracting responses on patient pain behaviors. This indicates that perceiving distracting responses from caregivers were negatively related to pain catastrophizing in patients (i.e., more distracting responses were related to less pain catastrophizing); in turn, pain catastrophizing was positively related to expressing pain behaviors (i.e., less catastrophizing was related to fewer pain behaviors).

#### Perceived Negative Responses as the Predictor and Pain Behaviors as an Outcome

Analyses showed that the total effect of perceived caregiver negative responses on pain behaviors (weight *c*) was significant, *F*(1,198) = 22.416, *p* < 0.001, *R*^2^ = 0.113, and the direct effect of perceived caregiver negative responses on pain behaviors (weight *c’*) was not significant [i.e., *b* = 0.631, *t*(198) = 4.734, *p* > 0.001]. In addition, the indirect effect (weight *a^∗^b*) was significant (coefficient = 0.036, 95%, CI = 0.020–0.057), indicating that patient pain catastrophizing plays a mediating role in the association between perceived caregiver negative responses and pain behaviors. This analysis indicates that perceiving the caregiver’s negative responses was positively related to expressing pain behaviors (i.e., more negative responses was related to more pain catastrophizing); in turn, pain catastrophizing was positively related to expressing pain behaviors (i.e., more catastrophizing was related to expressing more pain behaviors). Table 2 presents the results of the mediating analyses.

**TABLE 2:** Results of the mediation analyses with standard errors (SEs) and 95% confidence intervals.

Outcome of each step	Independent variable	Predictors	Coefficient	SE	*t*	*p*-Value	95% LL CI	95% UL CI
Pain catastrophizing	Mediation analysis with perceived caregiver solicitous responses as a predictor	Perceived caregiver solicitous responses (path *a*)	26.14	2.55	10.21	0.0000	21.09	31.19
Pain behaviors		Pain catastrophizing (path *b*)	0.06	0.0069	8.97	0.0000	0.04	0.07
Pain behaviors		Perceived caregiver solicitous responses (path *c*)	0.02	0.01	2.15	0.03	0.0023	0.05
Pain behaviors		Perceived caregiver solicitous responses (path *c*)	0.02	0.01	2.03	0.04	0.0008	0.04
		Indirect effect (a*b)	0.0046	0.0060*	−	−	−0.0076**	0.01**
Pain catastrophizing	Mediation analysis with perceived caregiver distracting responses as a predictor	Perceived caregiver distracting responses (path *a*)	–0.28	0.11	–2.45	0.01	–0.52	–0.05
Pain behaviors		Pain catastrophizing (path *b*)	0.06	0.0069	9.45	0.0000	0.05	0.07
Pain behaviors		Perceived caregiver distracting (path *c*)	0.0081	0.01	0.54	0.58	–0.02	0.03
Pain behaviors		Perceived caregiver distracting (path *c*)	0.02	0.01	2.26	0.02	0.0035	0.05
		Indirect effect (a*b)	–0.01	0.0081*	−	−	−0.03**	−0.0039**
Pain catastrophizing	Mediation analysis with perceived caregiver negative responses as a predictor	Perceived caregiver negative responses (path *a*)	0.63	0.13	4.73	0.0000	0.36	0.89
Pain behaviors		Pain catastrophizing (path *b*)	0.05	0.0078	7.38	0.0000	0.04	0.07
Pain behaviors		Perceived caregiver negative responses (path *c*)	0.06	0.01	3.73	0.0002	0.02	0.09
Pain behaviors		Perceived caregiver negative responses (path *c*)	0.02	0.01	1.49	0.13	–0.0084	0.06
		Indirect effect (a*b)	0.03	0.0096*	−	−	0.02**	0.05**

*p <.05; ** *p* <.01.

## Discussion

This study examined the mediating role of patient pain catastrophizing in the relationship between perceived caregiver responses (i.e., solicitous, distracting, and negative responses) and pain behaviors in patients with chronic pain. Our findings showed that perceived distracting response from caregivers is negatively related to lower levels of catastrophizing thoughts in patients, which in turn is positively related to expressing pain behaviors. This means that higher levels of perceived distracting responses from caregivers are related to a decreased level of pain catastrophizing thoughts in patients, which in turn is related to a decreased level of expressed pain behaviors. Interestingly, perceived more negative response from caregivers is related to higher levels of catastrophizing thoughts, which in turn is related to expressing more pain behaviors. We did not find any mediating role for pain catastrophizing in the relationship between perceived caregiver solicitous responses and expressed pain behaviors.

The current study showed that the relationship between perceived caregiver distracting responses and patients’ pain behaviors is mediated by patient pain catastrophizing. Specifically, it was found that higher levels of perceived caregiver distracting were related to lower levels of pain catastrophizing in patients, which were related to fewer pain behaviors in patients. This is in line with the nature of caregiver distracting responses, which aim to distract the patient’s attention from the pain and help them to engage in another activity (McCracken, 2005). It has been suggested that distracting strategies may not be effective in patients that tend to catastrophize their pain as they tend to be hypervigilant to any pain cues (Peters et al., 2002). Moreover, the effectiveness of distracting strategies is still in question. In some cases, it has been suggested that using these strategies is related to experiencing more intense pain and fatigue because performing distracting tasks demand effort (Johnson, 2005). However, the findings of this study do show that perceiving more caregiver distracting responses are related to less catastrophizing thoughts. This might indicate that while patients’ distracting strategies may not impact their attention to pain cues, when the distraction is coming from an external source (e.g., caregivers), this actually might be more beneficial. This is in line with the studies on children that have shown that parents’ distracting strategies can reduce pain and pain behaviors in their children (e.g., McCarthy et al., 2010).

The results also showed that perceived caregiver negative responses are related to higher levels of pain catastrophizing, which in turn is related to expressing more pain behaviors. This is similar to the findings of other studies that found negative responses by caregivers are associated with more negative outcomes in patients with chronic pain, including anxiety (Cano et al., 2004), depression, pain intensity (Cano et al., 2000), and pain behaviors (Leonard et al., 2006). It is likely that when patients perceive more negative and hostile responses from their caregiver, they realize that they would not receive the help and support that they need, which can induce helplessness and catastrophizing cognitions. Besides, when patients perceived negative and punishing responses, they may engage in expressing more behaviors in an attempt to convince their caregiver that their pain is real and, in an attempt, to elicit supportive responses from their caregiver (Leonard et al., 2006). Moreover, these findings are also in line with the expressed emotion theory, which indicates that high levels of hostility and criticism in a family environment contribute to vulnerability to stress (Faucett and Levine, 1991) and persistence of illness symptoms (Hooley and Gotlib, 2000; Hooley, 2007).

Additionally, in this study, it has been observed that while perceived caregiver solicitous responses were slightly and positively related to expressing pain behaviors, they were not related to pain catastrophizing, and pain catastrophizing did not mediate the link between perceived caregiver responses and pain behaviors. These results are partially in line with the studies that have shown that caregivers’ solicitous responses are positively associated with expressing more pain behaviors and, consequently, higher levels of disability (Romano et al., 1995; Boothby et al., 2004). However, it seems that the association between perceived caregiver solicitous and pain behaviors cannot be explained with pain catastrophizing, and as it was suggested by others, it still needs further investigation (Bernardes et al., 2017). For example, it is likely that when patients perceived high levels of solicitous responses, they also perceive a better relationship with their caregivers, and therefore, they feel more comfortable expressing their pain behaviors to express their pain. It is in line with the studies that indicated that solicitous responses are more reinforcing when couples are maritally satisfied (Leonard et al., 2006). In the current study, we did not investigate the relationship quality between our participants and their caregivers. Future studies need to investigate this relationship to have a better understanding of when and how solicitous responses impact catastrophizing cognitions.

When interpreting the result of this study, some limitations need to be taken into account. First, this study is only based on patients’ perceptions of their caregivers’ responses. While it is the patients’ cognitions and perceptions of their caregivers that impact their responses and behaviors than how caregivers respond to them, it is still important to investigate the responses to pain behaviors based on caregivers’ reports. Future studies may benefit from the comparison of patients’ understanding of caregivers’ reports and caregivers’ reports. Second, this is a cross-sectional study, and therefore, it is impossible to assess the causal effect. Third, we set limited inclusion and exclusion criteria to prevent problems associated with the selective sampling of participants, but it resulted in heterogeneity in data and influenced the generalizability of the findings. Future studies are advised to be more exclusive in inclusion criteria to improve the generalizability. Besides, only participants with chronic musculoskeletal pain were recruited in this study, which might limit the generalizability of the findings to patients with other types of pain (e.g., cancer pain). Finally, some factors, such as relationship quality, have not been investigated in this study, which can play an important role in understanding how caregivers’ responses impact patients’ cognitions.

Despite the above-mentioned limitations, this study has an important clinical implication. The results show that patients’ perceptions of their caregivers’ responses impact their cognitions and also related to how they express their pain. Therefore, when it comes to pain treatment and developing interventions for these patients, it is important to evaluate these perceptions and invite the caregivers to the intervention sessions to help the caregivers understand the meaning and impact of their responses on their patients with chronic pain. Besides, helping patients to have a better understanding of how external sources can impact their thoughts and behaviors can also increase the level of control over their pain management and pain coping strategies.

## Data Availability Statement

The datasets generated for this study are available on request to the corresponding author.

## Ethics Statement

The studies involving human participants were reviewed and approved by the ethics committee of the Department of Psychology at Shahid Beheshti University. The patients/participants provided their written informed consent to participate in this study.

## Author Contributions

SM was involved in analysis and writing. FA was involved in data acquisition, analysis, and writing. NE was involved in data acquisition and writing. MD and AK were involved in the design, data acquisition, analysis, and writing. All authors contributed to the article and approved the submitted version.

## Conflict of Interest

The authors declare that the research was conducted in the absence of any commercial or financial relationships that could be construed as a potential conflict of interest.
